# Can different stages of leprosy treatment influence the profile of oral health? Oral status in leprosy

**DOI:** 10.4317/medoral.22220

**Published:** 2018-06-21

**Authors:** Fernanda-Zanol Matos, Andreza-Maria-Fábio Aranha, Álvaro-Henrique Borges, Fábio-Luiz-Miranda Pedro, Suzane A. Raslan, Fádua Hamida, Kadyja Veiga, Alessandra-Nogueira Porto

**Affiliations:** 1 Post graduate of the postgraduate program of the University of Cuiabá- Mato Grosso; 2 PhD Professor of the Postgraduate Course of Dentistry - University of Cuiabá- Mato Grosso; 3 Professor at UNIVAG-Várzea Grande Undergraduate Course - Mato Grosso; 4 Academic of Dentistry - University of Cuiabá- Mato Grosso

## Abstract

**Background:**

The aim of study was to evaluate the oral health status, salivary flow and halitosis among individuals diagnosed with leprosy as compared with healthy subjects.

**Material and Methods:**

A sample of 160 individuals was allocated into four groups, as follows: (G1) individuals with complete leprosy treatment; (G2) individuals diagnosed with leprosy and under multi-drug therapy; (G3) individuals diagnosed with leprosy not yet under treatment; and (G4) healthy individuals. Then individuals were submitted to periodontal clinical examination (visible plaque index, bleeding index, depth of probing and clinical attachment level); DMFT index (decayed-missing-filled teeth index); evaluation of salivary flow and halitosis using a halimeter equipment (Interscan Corp, Chatsworth, CA, USA).

**Results:**

The data were analyzed using Kruskal-Wallis and chi-square tests. The mean DMFT was found to be higher than 6.6, which is considered very high, with no significant difference between groups (*P*>0.05). As for salivary flow, 76.2% of the subjects presented normal flow rates, while 10% and 13.7% showed low and very low salivary flow rates, respectively, with hyposalivation being mostly observed in Groups 1 and 2. The highest prevalence of noticeable odor was found in healthy individuals (G4), and the most prevalent periodontal diagnosis was gingivitis (63.1%) in Group 3 (individuals with leprosy not yet under multi-drug therapy) followed by periodontitis (25%) in Group 1 (individuals who had completed leprosy treatment).

**Conclusions:**

It was observed that individuals with a history of leprosy present poor oral health similar to that of systemically healthy individuals.

** Key words:**Leprosy, Periodontal diseases, Halitosis, CPO Index.

## Introduction

Leprosy is a chronic granulomatous infectious disease caused by *Mycobacterium leprae*, which presents with slow evolution with skin, peripheral nerve, and mucous membrane tropism (especially the respiratory tract) and has high infectivity and low pathogenicity (1).

Mato Grosso, located in the Midwest region of Brazil, is part of the Brazilian Amazon. This region has high leprosy detection rates and was classified, according to the parameters of the Ministry of Health ordinance MS/GM N. 3125 of October 7, 2010, as hyperendemic. This region has the highest prevalence of any Brazilian state: 10.19 cases/10,000 inhabitants (2,3).

The clinical forms of leprosy are classified according to the interactions of *M. leprae* with the host immune response (2). The Madrid classification consists of clinical and bacilloscopic characteristics, dividing leprosy into two immunologically unstable groups, i.e., indeterminate and borderline, and two stable polar types, i.e., tuberculoid and lepromatous. The Ridley & Jopling characterisation is based on clinical, bacilloscopic, immunological and histopathological criteria (1-3).

In 1982, the World Health Organization (WHO) proposed a simplified classification based on clinical manifestations and bacilloscopy in which cases with five or fewer skin lesions were considered paucibacillary (PB), whereas those with more than five skin lesions were considered multibacillary (MB) (2). This determination was crucial for the selection of drug therapy, and it reduced misdiagnoses, disease reactivation and even secondary resistance (2).

The upper airways are the most important gateway to the bacillus and the main source for bacillary elimination in leprosy. The oral mucosa is likely the second major site of *M. leprae* infection and transmission, and it plays a fundamental role in the transmission of adult leprosy to children. Several anatomic sites affected by leprosy and the most commonly affected regions of the oral cavity are the hard palate, soft palate, uvula, lips, tongue, and gingiva as well as the anterior region of the maxilla, which shows significant bone erosion and dental loss (3,4).

Periodontal diseases (PDs) are highly prevalent among adults. PDs consist of an inflammatory process of infectious origin, wherein the dental biofilm plays an important role in the process of pathogenicity, causing the destruction of the tooth support tissue. In addition to the appearance of periodontal pockets, insertion loss, inflammation, and the degradation of soft tissues, halitosis is a consequence of PD. The intraoral etiological factors of bad breath are associated with the presence of tongue coating in 85% to 90% of cases (5).

Halitosis is a breath alteration condition that is unpleasant for both the patient and those with whom the patient interacts. Because a social life is one of the pillars of quality of life, it is necessary to consider halitosis as a factor of negative interference that might denote a pathological condition (4,5).

Currently, caries and its consequences (obturation and tooth loss) are pathologies that are highly prevalent in the population and affect individuals across different age groups. Thus, caries has been a constant concern of the institutions responsible for evaluating and promoting measures to improve population health. The decay, missing, filled (DMF) index has been widely used in epidemiological surveys of oral health. The World Health Organization (WHO) recommends that this index be used to measure and compare the dental caries experience within populations. Its value expresses the average number of decayed, lost, and obturated teeth in a group of individuals (6,7).

Many factors, such as salivary gland origin, diet, the use of pharmacological agents, and the oral and systemic health of the individual, might affect composition and salivary flow (8).

Some of the several side effects of polychemotherapy (PCT) for leprosy include dry skin and mucous membranes, gastrointestinal disorders (e.g., abdominal and epigastric pain, diarrhea, nausea, vomiting, gastrointestinal intolerance), and changes in excretions (e.g., urine and feces) and secretions (e.g., sweat and saliva) (9,10).

A variety of oral changes have been associated with the hypofunction of the salivary glands, including increased dental caries, PD, mucositis, angular cheilitis, and an altered sense of taste. Most efforts to diagnose salivary gland hypofunction include salivary flow measurements (11).

Thus, despite the literature demonstrating the oral health profile of patients with leprosy through DMF indices, a significant gap exists in the knowledge concerning the oral profile before, during, and after treatment using PCT. Therefore, the current research evaluated oral health condition, salivary flow, and halitosis across the different stages of leprosy treatment and compared the results with those of healthy individuals.

## Material and Methods

The present observational study was approved by the Ethics and Research Committee of the University of Cuiabá (Process n. 281.921) in compliance with Resolution 196/96 of the National Health Council. Only the eligible individuals who agreed and signed the Free and Informed Consent Document participated in this study. The populations of this study consisted of individuals diagnosed with leprosy and treated at the Júlio Muller University Hospital in Cuiabá, Mato Grosso, Brazil and systemically healthy individuals from the primary healthcare units of Cuiabá (MT, Brazil). Individuals were eligible for this study if they were over 18 years of age and not pregnant. For inclusion in the diagnosed but untreated group, participants should not have started medication. Individuals with mouth opening limitations preventing clinical examination, those who were immunosuppressed (HIV or transplanted), and those without a definitive diagnosis of leprosy were excluded from this study.

The convenience-based sample was composed of 160 individuals divided into four groups: Group 1 was composed of individuals who had already completed leprosy treatment (n = 40); Group 2 was composed of individuals with leprosy receiving PCT (n = 40); Group 3 was composed of individuals diagnosed with leprosy who had not begun treatment (n = 40); and Group 4 was composed of healthy individuals (n = 40). The following individual data were recorded: sex, age, family income, oral hygiene habits, and self-perception of oral health. The individuals were randomly selected based on the demands of the hospital and health unit.

-Clinical Evaluation

The intraoral physical examination was performed without prior prophylaxis or supervised brushing. To establish a diagnosis, the participants underwent complete periodontal examination with the aid of a mouth mirror and a manual periodontal probe (PCPUNC 15 Hu-Friedy®, Mfg Co Inc. Chicago, IL, USA), and the data were recorded in a periodontal file. The clinical parameters evaluated included the visible plaque index (VPI) and gingival bleeding index (GBI). Both the VPI and GBI are dichotomous examinations; the first examination checks for the presence of dental plaque, and the second checks for gingival inflammation (12). Probing depth (PD) and clinical insertion level (CIL) were evaluated in mm at six dental sites: mesiobuccal, buccal, distobuccal, mesiolingual, lingual, and distolingual. The third molars were not evaluated. PD refers to the distance in mm between the gingival margin and the bottom of the gingival groove/pocket. The CIL is the distance in mm between the cemento-enamel junction and the bottom of the gingival groove/pocket.

After the evaluation of the clinical parameters, a periodontal diagnosis was established: periodontal health denoted < 30% of the periodontal sites presented with gingival bleeding; gingivitis denoted > 30% of the periodontal sites presented with gingival bleeding; periodontitis denoted the presence of four or more teeth with one or more sites with PD ≥ 4 mm and with a loss of clinical insertion of ≥ 3 mm at the same site (12).

The DMF index was used to record elements classified as decay (code 1), filled (code 2), or missing (code 3) teeth that were indicated for extraction (code 4). Healthy teeth (code 5) were also classified. Subsequently, all of the elements involved were summed and divided by the number of individuals examined (7).

A single examiner previously calibrated according to the methodology described by Araújo *et al.* (13), obtained each clinical parameter. The standard error of the measurement (SEM) was used to describe the continuous variable (i.e., PD), and the Kappa test was used for the categorical variables (i.e., VPI, GBA, and DMF). Thus, ten tests were repeated within 30 days and submitted to analysis. The examiner was considered as calibrated based on the following scores: SEM ≤ 0.8 as well as K > 0.8 and < 0.95.

-Salivary Flow

Salivary flow was evaluated via the stimulation of saliva with a block of Parafilm® (Bemis NA, Chicago, IL, USA) and measured according to the recommendations of Flink *et al.* (14). After receiving a 20-ml wide-mouth vial, participants sat relaxed in front of the examiner, keeping their mouth open and their tongue positioned on the palatal surface of the upper incisors. Then, participants chewed Parafilm® for 2 minutes to stimulate salivation. Next, they spit all of the saliva formed into the vial. At exactly 5 minutes (timed by the examiner), the patient spat for the last time. The total volume of saliva collected in 5 minutes was checked, excluding foam. The final value was divided by five, and the salivary flow result obtained in ml/min was recorded in the clinical file. The stimulated salivary flow rate was considered as normal for values between 1.0 and 3.0 ml/min (15).

-Evaluation of Halitosis

Halitometry was performed using the Halimeter® (Interscan Corp, Chatsworth, CA, USA) apparatus in which a disposable straw was connected to the device and placed approximately 4 cm in the posterior region of the mouth to evaluate the presence or absence of volatile compounds with a strong odor.

The patient remained with semi-open lips, without breathing for 15 seconds, and the value displayed on the equipment during the maximum peak was recorded, which represented the concentration of oral volatile sulfur compounds (VSCs). The individuals then exhaled their pulmonary air to detect the presence of systemic VSCs (16).

The VSC results were interpreted according to the manufacturer’s instructions: less than 80 ppb denoted no perceptible odor, 80 to 100 ppb denoted perceivable odor, 100 to 120 ppb denoted moderate halitosis, 120 to 150 ppb denoted more pronounced halitosis, and > 150 ppb denoted severe halitosis.

## Results

The individuals evaluated were between 18 and 78 years old, and Group 1 (individuals who already completed leprosy treatment) showed a higher mean age (48.9 years), followed by Group 2 (leprosy patients receiving PCT; 45.7 years), Group 3 (individuals diagnosed with leprosy who had not begun treatment; 44.1 years), and Group 4 (healthy individuals; 37.3 years). Of these patients, 57.5% were men, and 41.2% were mestizo, followed by 38.1% black and 20.7% white (*p* = 0.0386, chi-square test). Education, 42.5% had completed between the 5th and 9th grade. The results demonstrated significant inter-group differences (*p* < .0001, chi-square test). Additionally, socioeconomic status, 59.3% of the individuals reported receiving from 1 to 3 minimum wages within their families. One hundred and ten cases met the operational classification for multibacillary leprosy (91.6%) and ten paucibacillary (8.4%).

Regarding tobacco use, 56.8% of the participants reported not being smokers, and 21.8% reported being ex-smokers. Notably, oral hygiene 91.2% of respondents said that they brushed their teeth, and 41.2% said they brushed three or more times per day. The flossing, 60% did not floss; however, of those who reported flossing, 20% reported flossing twice a day. Tongue hygiene, 75% reported brushing their tongues. However individuals’ self perceptions of oral problems, 62.5% said they did not feel or have bad breath, 48.1% stated that they did not perceive halitosis. At the time of the present study, the presence of gingival bleeding, 54.3% reported no bleeding, 23.2% reported that their gums bled, and 22.5% stated that sometimes the gingiva presented with bleeding ( Table 1).

**Table 1:**
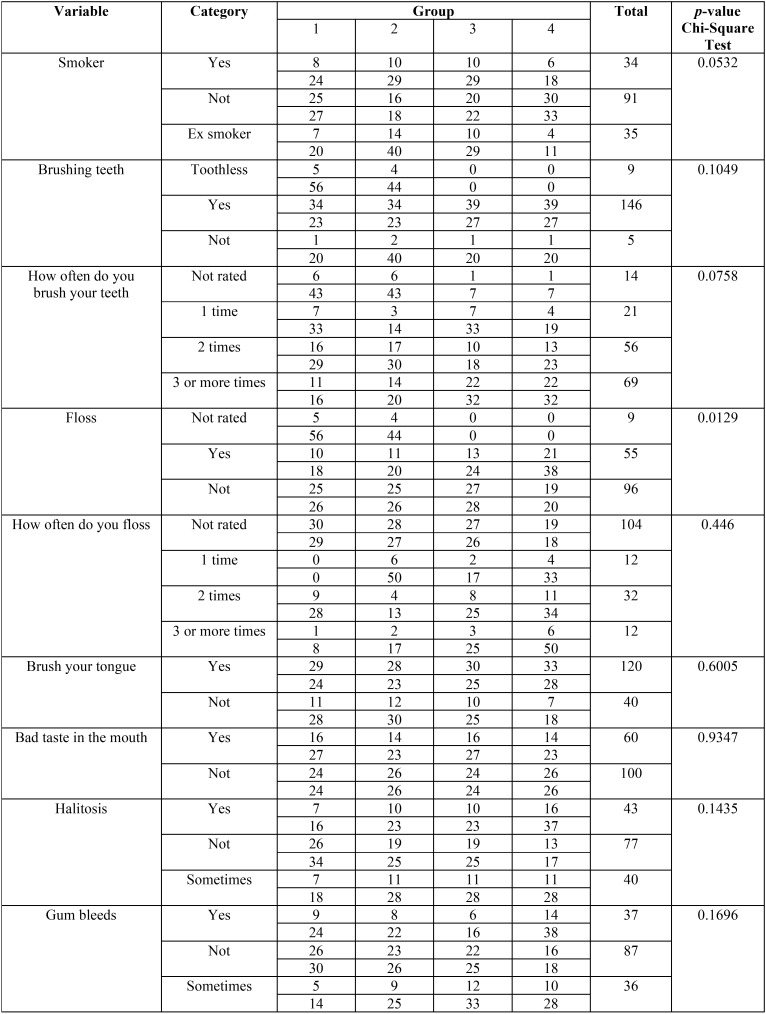
Habits and perception of oral health of groups of individuals diagnosed with leprosy and control group.

In the dental clinic evaluation, the mean DMF index score was > 6.6 for all groups, which is considered high according to the Ministry of Healthd. Group 1 had the highest mean DMF (17.05), followed by Group 4 (16.3); however, Group 2 (14.8) and Group 3 (14.2) presented with approximately equal values. Thus, the inter-group evaluation did not reveal significant differences (*p* > 0.05). The evaluation of salivary flow, 76.2% presented with normal salivary flow, 10% of the individuals had low salivary flow, and 13.7% had very low salivary flow. The individuals who presented with the most hyposalivation belonged to Groups 1 and 2. The results showed significant differences (*p* = 0.0012) between groups ( Table 2).

**Table 2:**
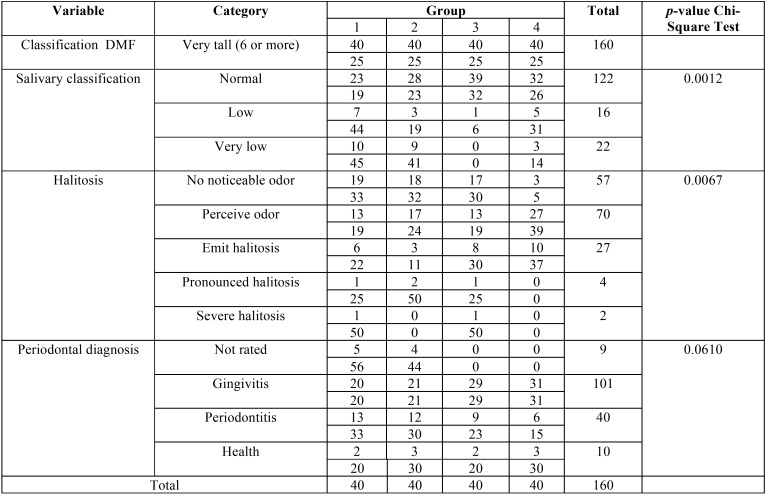
Classification of DMF, salivary, halitosis and periodontal diagnosis of the groups of individuals diagnosed with leprosy and control group.

For the halitosis test, 43.7% of the individuals presented with a noticeable odor, 35.6% presented with no noticeable odor, 2.5% presented with pronounced halitosis, and 1.2% had severe halitosis. A high concentration of Group 4 individuals had halitosis, and a significant difference (*p* = 0.0067) was observed between groups ( Table 2).

In the periodontal clinical diagnosis, 63.1% of the individuals presented with gingivitis, 25% presented with periodontitis, and 6.2% showed gingival health. No significant difference (*p* > 0.05) was found between groups ( Table 1). About the periodontal clinical parameters GBI, VPI, CIL, and PD, the Kruskal-Wallis test used for intergroup analysis did not reveal a significant difference (*p* > 0.05;  Table 3, Table 4).

**Table 3:**
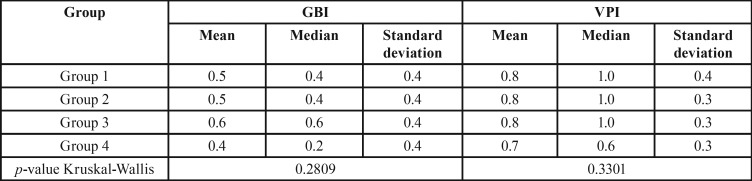
Mean, median and standard deviation of GBI and VPI per group.

**Table 4:**
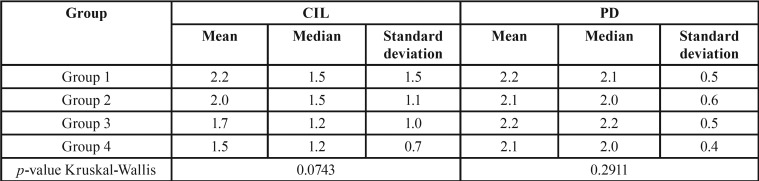
Mean, median and standard deviation of CIL and PD by group.

## Discussion

Leprosy is an age-old disease that affects modern populations mainly located in India and Brazil. This disease continues to have a significant global impact, with official reports from 121 countries. According to the WHO, 213,899 newly diagnosed cases were recorded in 2014 (17).

The present study observed a mean participant age of 44.1 years. At this age, the individual is considered as economically active and productive, and the sequelae of leprosy due to late diagnosis can affect the lives of these individuals, isolating them from social interactions and making it impossible for them to exercise their normal work activities because of upper limb atrophy, which might even cause oral hygiene limitations (18).

The present study identified a predominance of mestizo individuals in all groups (41.2%), which corroborates the results of other studies (19,20).

Concerning the education level, the majority (42.5%) had completed elementary school. Furthermore, to income, the study showed a predominance of one to three minimum wages in all groups. According to some authors, populations with lower socioeconomic statuses have difficulty accessing dental services, which leads to poorer dental conditions and possible outbreaks of oral cavity infections (19,20). The transmission of leprosy is influenced by socioeconomic conditions, nutritional status, genetic conditions, and concomitant infections (21).

No significant difference between groups was found tobacco use; most of the individuals, regardless of group, were non-smokers, and the smoker distribution was homogeneous. Although smoking is a risk factor for PD and favors its progression, in the present study, this variable did not show a specific negative interference for any group, regardless of the diagnosis of leprosy. This response is most likely related to the small number of smokers (22).

Concerning to oral hygiene, dental brushing, and how many times a day the individual brushes his or her teeth, the majority of individuals within the studied groups reported brushing their teeth three times a day or more. However, the quantity of brushing should be associated with its quality (23), and the present study negatively verified this result given the high rate of PD and consequent halitosis (noticeable odor and moderate halitosis), even among the systemically healthy group. A significant difference was observed for dental flossing (*p* = 0.0129) in which most individuals did not use dental floss; when they did, they reported using it twice a day or more, regardless of the group studied. Although the systemically healthy group flossed more frequently than the other groups, the non-use of dental floss is justified by its financial (socioeconomic conditions) and cultural (education) associations.

Concerning tongue cleanliness, the majority (75%) of individuals reported cleaning their tongues; however, other studies have reported that the efficient cleaning of the tongue daily reduces halitosis through microbial reduction on its surface via a toothbrush or scrapers (5,8), Although individuals reported tongue cleaning in the present study, the high halitosis results indicate a deficiency in this cleaning.

A lack of self-perceptions of oral health was verified by the most of the individuals (62.5%) reported no perception of bad taste in the mouth, bad breath (48.12%), or gingival bleeding (54.37%). Thus, the perception and knowledge of the individual upon what is pathological or physiological is somewhat complex because PD has a slow and progressive evolution. Often, the individual feels that a bad taste in the mouth or bad breath are personal traits or that result from gastric problems. Gum bleeding is also believed to result from harder foods or mechanical trauma due to the incorrect use of a toothbrush or floss (24-26).

Similar to the average found by Núnez-Martí *et al.*, all groups presented a high score of DMF index (> 6.6), and no significant difference was found between groups of individuals diagnosed with leprosy and healthy individuals with regard to DMF (27).

In the present study, periodontal condition was exhibited in all individuals of groups with gingivitis (63.1%); periodontitis (25%) and only 6.25% presented with gingival health. These results corroborate those of Souza *et al.* (24), who used a periodontal clinical examination to diagnose approximately 80 to 88% of patients with PD. When comparing the results of patients diagnosed with leprosy and healthy individuals, the findings were similar: Both presented with poor oral health status, a finding that was also observed in the present study.

Approximately 43.7% of the individuals presented a noticeable odor on the halitosis test. Strangely, a higher prevalence of halitosis (noticeable odor) was observed in Group 4, even in a normal flow of saliva, however this result is justified by the presence of dental biofilm found in the majority of this group presented with gingivitis, moreover an oral microbiota not altered by the non use of medicines, in case polychemotherapies and in agreement with some authors (5,9,11), no severe or pronounced halitosis was found in Group 4. Halitosis may occur

as a consequence of decreased oral clearance due to bleeding or as a result of dental and oral mucosal disease (5,16).

Severe or pronounced halitosis was verified in Groups 1, 2, and 3, which were related to leprosy and coincided with low and very low salivary flows in Groups 1 and 2, respectively. This fact raises a question about the capacity of the drugs ingested to treat leprosy (dapsone, rifampicin, and clofazimine) to alter (modifiy) salivary flow, in addition to systemic and local changes, might manifest side effects in the oral cavity according to Femiano *et al.* wich reported that more than 500 medications that are currently in use produces sides effects; however, in the majority of cases, the mechanisms are unknown (5,6,11,28).

The present study excluded the relationship between halitosis and the destruction of oral structures (e.g., perforation of the palate) as a consequence of leprosy. This finding leads us to believe that leprosy campaigns have helped to avoid its late diagnosis and advanced stages of the disease.

Many studies have examined the diagnosis and treatment of leprosy, but few have addressed oral involvement in leprosy. Cortella *et al.* (29) and Fucci da Costa *et al.* (23) stated that the oral cavity inflammatory process, either via dental infection or PD, can lead to leprosy reactions that are acute inflammatory processes secondary to the release of antigens and hypersensitivity reactions. These reactions are extremely deleterious and can lead to irreversible sequele among patients with leprosy.

Given the magnitude of leprosy with regard to public health and the high prevalence of the disease in Brazil, it should be discussed more often in dentistry courses because the upper airways are the most important entryway of the bacillus, and the face is a location of broad disease onset.

The evaluation of the oral condition of individuals diagnosed with leprosy and healthy individuals allowed us to conclude the following:

•The DMF index was high for all groups, with a higher prevalence in Group 1 (individuals who had already completed leprosy treatment).

•Salivary flow was normal in 76.2% of individuals, with a higher prevalence of hyposalivation in Groups 1 (individuals who had already completed leprosy treatment) and 2 (patients with leprosy receiving PCT). This finding leads us to consider the possible influence of a medication such as PCT on salivary flow.

•Concerning halitosis, noticeable odor showed the highest prevalence (43.7%) in Group 4 (healthy individuals), with a significant difference between groups (*p* = 0.0067).

•In the periodontal diagnosis, most individuals presented with some type of PD, with gingivitis being the most prevalent (63.1%) in Group 3 (individuals with leprosy who did not begin treatment) and periodontitis the most prevalent (25%) in Group 1 (individuals who had already completed the leprosy treatment).
